# Genome-Wide Association Analysis Reveals Genetic Architecture and Candidate Genes Associated with Grain Yield and Other Traits under Low Soil Nitrogen in Early-Maturing White Quality Protein Maize Inbred Lines

**DOI:** 10.3390/genes13050826

**Published:** 2022-05-05

**Authors:** Olatunde A. Bhadmus, Baffour Badu-Apraku, Oyenike A. Adeyemo, Paterne A. Agre, Offornedo N. Queen, Adebayo L. Ogunkanmi

**Affiliations:** 1 Department of Cell Biology and Genetics, University of Lagos, Lagos 101017, Nigeria; oladotunbhadmus@gmail.com (O.A.B.); aoadeyemo@unilag.edu.ng (O.A.A.); adebayoogunkanmi@yahoo.com (A.L.O.); 2 International Institute of Tropical Agriculture, IITA, PMB 5320 Oyo Road, Ibadan 200285, Nigeria; p.agre@cgiar.org (P.A.A.); o.queen@cgiar.org (O.N.Q.)

**Keywords:** low-N, GWAS, candidate genes, QPM, marker-assisted selection

## Abstract

Maize production in the savannas of sub-Saharan Africa (SSA) is constrained by the low nitrogen in the soils. The identification of quantitative trait loci (QTL) conferring tolerance to low soil nitrogen (low-N) is crucial for the successful breeding of high-yielding QPM maize genotypes under low-N conditions. The objective of this study was to identify QTLs significantly associated with grain yield and other low-N tolerance-related traits under low-N. The phenotypic data of 140 early-maturing white quality protein maize (QPM) inbred lines were evaluated under low-N. The inbred lines were genotyped using 49,185 DArTseq markers, from which 7599 markers were filtered for population structure analysis and genome-wide association study (GWAS). The inbred lines were grouped into two major clusters based on the population structure analysis. The GWAS identified 24, 3, 10, and 3 significant SNPs respectively associated with grain yield, stay-green characteristic, and plant and ear aspects, under low-N. Sixteen SNP markers were physically located in proximity to 32 putative genes associated with grain yield, stay-green characteristic, and plant and ear aspects. The putative genes *GRMZM2G127139, GRMZM5G848945, GRMZM2G031331, GRMZM2G003493, GRMZM2G067964, GRMZM2G180254*, on chromosomes 1, 2, 8, and 10 were involved in cellular nitrogen assimilation and biosynthesis, normal plant growth and development, nitrogen assimilation, and disease resistance. Following the validation of the markers, the putative candidate genes and SNPs could be used as genomic markers for marker-assisted selection, to facilitate genetic gains for low-N tolerance in maize production.

## 1. Introduction

The global demand for maize is expected to double by the year 2050 [1]. According to Shiferaw et al. [2], more than half of the demand for cereals by 2050 will come from maize. This represents more than 70% of the present world food demand. To meet this demand approaches that will facilitate accelerated genetic gains in the genetic enhancement of maize are required [3]. However, efforts towards crop improvement based on conventional plant breeding tools are limited and time consuming for selecting genotypes with desirable traits [3,4,5].

Nitrogen is an essential macronutrient that is required for the optimum growth and development of the maize plant, but it is the most limiting in SSA soils. It is an essential constituent of chlorophyll, the green pigment required for photosynthesis in leaves [6]. It plays a significant role in plant metabolism, and it is also an important constituent of protein synthesis and of nucleic acids [7]. The lack of nitrogen at the physiological stage in maize development reduces leaf area development and photosynthetic rate, resulting in kernel and ear abortion, and accelerates leaf senescence and reduces crop kernel weight [8,9]. The edaphic condition of nitrogen-depleted tropical soils is further aggravated by the subsistence level of resource-poor smallholder farmers who can hardly afford the high cost of mineral fertilizers to supplement the low nitrogen in the soil. Consequently, maize production in the sub-region is critically affected, either by inadequate use and the high cost of nitrogen-based fertilizers, or the non-availability of fertilizers or lack of funds to farmers [9,10,11]. The lack of nitrogen in the soil may lead to yield reductions of more than 70% under severe low-N stress conditions [12,13]. The development of low-N tolerant maize genotypes remains an effective strategy for ensuring increased maize productivity on nitrogen-depleted soils [14,15,16].

The advent of genomics and the identification of genomic regions underlying the inheritance of important traits have helped to improve the efficiency of selection processes in crop improvement programs [17,18]. GWAS has been carried out in maize to identify QTLs associated with important traits, including aflatoxin resistance, heat stress, and drought tolerance; *Fusarium* ear rot, as well as *Striga* resistance [19,20,21,22,23]. López-Malvar et al. [24] conducted a GWAS study using a subset of 408 recombinant inbred lines to identify SNPs associated with yield and saccharification efficiency in maize stover. They identified 13 SNPs to be significantly associated with increased stover yield, and 2 SNPs that were significantly associated with improved saccharification efficiency. Adewale et al. [22], in a GWAS study in maize, also identified 24 SNPs that were significantly associated with *Striga* resistance, including grain yield, *Striga* damage at 8 and 10 weeks after planting, ears per plant, and ear aspect. Additionally, a GWAS study by Ertiro et al. [25] identified 45 SNPs that were significantly associated with grain yield, plant height, ear height, ear position, ears per plant, and senescence traits, using 424 CIMMYT maize inbred lines evaluated under both optimum and low-nitrogen conditions. Similarly, He et al. [26] identified 50 significant SNPs associated with nitrogen use efficiency-related traits, such as N uptake efficiency, N utilization efficiency, grain N concentration, stover N concentration, and N harvest index, under optimal nitrogen and low-nitrogen conditions. However, there is a need to identify SNP markers that are associated with grain yield and other low-N-tolerance adaptive traits used for the selection of tolerant maize genotypes in the International Institute of Tropical Agriculture Maize Improvement Program (IITA-MIP). Additionally, these traits have been found to be significantly correlated with grain yield, and they are widely used in low-N studies in maize in SSA [12,16,27,28,29,30,31,32]. The identification of SNP markers associated with these traits would facilitate the rapid selection of maize genotypes, through the application of marker-assisted selection (MAS) in maize breeding research in SSA. The objectives of this study were to (i) determine the genetic structure of 140 early-maturing white QPM maize inbred lines with varying levels of tolerance to low-N, and (ii) identify the genomic regions and the marker trait-associated SNP markers, as well as the putative candidate genes associated with grain yield and other agronomic traits under low-N.

## 2. Materials and Methods

### 2.1. Plant Materials

One hundred and sixty-nine tropical (169) QPM inbred lines used in this study were extracted from the F_1_ maize hybrids of 10 bi-parental crosses involving crosses among extra-early white QPM inbred testers and early-maturing white QPM inbred testers. The QPM inbred line testers were identified to have positive and significant general combining abilities from previous studies [9,30]. The F_1_ hybrids were taken through a cycle of backcrossing to the extra-early inbred testers to recover the earliness. The BC_1_F_1_ with desirable agronomic characteristics were selected using the pedigree selection method from each backcrossed population, and advanced through repeated inbreeding to the S_9_ generation (Table S1) (Figure S1).

### 2.2. Phenotyping

The 169 QPM inbred lines were evaluated using a 13 × 13 lattice design with two replications, under low-N conditions at Mokwa (9°18′ N, 5°4′ E, 457 m altitude, 1100 mm annual rainfall) during the 2019 and 2020 rainy season in Nigeria. The low-N experiment was carried out at Mokwa where the soil had been depleted of nitrogen by the continuous growing of maize without the application of fertilizer, and by the removal of the biomass after each cropping season. Therefore, the low-N blocks were assumed to have been depleted of nitrogen to a level of zero. During each season, three seeds were planted per hill, and seedlings were thinned to two plants per hill, 2 weeks after planting (WAP), to obtain a target population density of 66,666 plants ha^−1^. The seeds were planted in single-row plots 3 m long, with a spacing of 0.75 m, and 0.40 m between and within rows, respectively. Nitrogen fertilizer in the form of urea (30 kg N ha^−1^) and 15 kg N ha^−1^ were applied at 2 WAP, with an additional 15 kg N ha^−1^ applied at 4 WAP. The low-N plots received 60 kg ha^−1^ each of single superphosphate (P_2_O_5_) and muriate of potash (K_2_O) at 2 WAP. The low-N plots were kept weed-free via the application of atrazine and gramozone as pre- and post-emergence herbicides at 5 L/ha, respectively, and subsequently supplemented with hand weeding to keep the plots weed-free. Under low-N conditions, data were collected for plant aspect (PASP) on a scale of 1 to 9, based on normal plant growth and appeal to sight, where 1 = excellent plant growth and 9 = extremely poor growth. The ear aspect (EASP) assesses freedom from disease and insect damage; ear size and the uniformity of ears was scored on a scale of 1 to 9, where 1 = large, uniform, clean, and well-filled ears and 9 = small, dirty, and poorly filled ears. The stay-green characteristic (STGR) was assessed on a scale of 1 to 9, where 1 = all leaves were green, and 9 = all leaves were dead. 

### 2.3. Phenotypic Data Analysis

The analysis of variance (ANOVA) was performed for the test environments for grain yield and other agronomic traits with PROC GLM in SAS [33], using a RANDOM statement with the TEST option. The combination of year and location was considered to be an environment. The phenotypic data across the environment were converted to a single best linear unbiased estimate (BLUE) value, using the linear mixed model in META—R [34]:

$$Y_{IJKL\;}=\mu +B{\left(E\right)}_{J\left(i\right)}+G_{k}+GE_{ij}+e_{ijkl}$$

where, *Y_IJKL_* = phenotypic observation for a trait, *µ* = grand mean, *E* = environment effect (location), *B(E)* = replication effect nested in a location, *G* = genotype effect, *GE* = genotype by environment interaction, *e* = random residual error. The correlation analysis was performed using the performance analytics package in R [35]. The broad sense heritability (H^2^) estimates were calculated from the phenotypic variance ($\sigma_{p}^{2}$) and the genotypic variance ($\sigma_{g}^{2}$) [36]:

$$H^{2}=\frac{\sigma_{g}^{2}}{\sigma_{g}^{2}+\frac{\sigma_{e}^{2}}{r}\;}$$

where $\sigma_{g}^{2}$ is the variance attributable to the genotypic effects, $\sigma_{e}^{2}$ is the experimental error variance; and r is the number of replicates within each environment. 

### 2.4. DNA Extraction and Genotyping

Even though leaf samples were collected from the 169 QPM inbred lines, due to funding limitations, leaf samples from only 140 QPM inbred lines were collected for DNA extraction. Genomic DNA was isolated from the freeze-dried leaf tissues following the modified Cetyl-trimethyl ammonium bromide (CTAB) protocol as described by Azmach et al. [37]. The DNA quality and quantity were ascertained using the UV absorbance protocol in the FlUOstar Omega microplate reader (BMG LABTECH). The extracted genomic DNA samples were sent for high-density whole-genome profiling by Diversity Arrays Technology sequencing (DArTseq), DArT Pty Ltd., Australia (https://www.diversityarrays.com, accessed on 27 August 2021), following the protocol described by Jaccoud et al. [38]. Reads and tags found in each sequencing result were aligned to the *Zea mays* L. genome reference, version B73V4 (B73 Ref-Gen v4 assembly) [39], which provided a raw dataset of 49,185 DArTseq markers. The 49,185 DArTseq markers were filtered to eliminate SNPs with missing values >10%, a heterozygosity >20%, and a minor allele frequency (MAF) < 5%. SNPs with unknown chromosome position were also eliminated. After quality filtering, a total of 7599 DArTseq markers distributed across the 10 maize chromosomes were retained for future analysis.

### 2.5. Population Structure and Linkage Disequilibrium (LD)

Population structure analysis was conducted to assess the population subgroups, using structure software version 2.3.4 [40,41]. Structure simulations were carried out using an admixture model with a burning period of 30,000 iterations and a Markov chain Monte Carlo (MCMC) set at 30,000. The simulations were repeated three times for K-values of 1 to 10. The subpopulation model was investigated in several ways by considering ΔK, a second-order rate change with respect to K, as defined by Evanno et al. [42], and as implemented in STRUCTURE HARVESTER [43]; thus, the most likely value of K was determined. The structure population was then plotted using the barplot function implemented in R. The phylogeny tree was constructed using the *Ape* package in R [44]. The MAF and the observed and the expected heterozygosity, as well as the polymorphism information content, were estimated using the function “--freq” and “--hardy” in PLINK V1.90 [45]. The genome-wide linkage disequilibrium (LD) was estimated as a squared allele frequency correlation coefficient (r^2^) between all possible pairs of SNPs using PLINK [45]. The LD decay rate was estimated by plotting the r^2^ values versus the corresponding physical distances between the SNP pairs, using the GAPIT R package [46].

### 2.6. Genome-Wide Association Analysis

A compressed mixed linear model (CMLM) implemented in the Genome Association and Prediction Integrated Tool (GAPIT) R package was used to compute associations between the SNPs and traits, using the mixed model proposed by Yu et al. [47]. The model considered the molecular markers as fixed effects, and were evaluated individually: *Y = Xβ + Wα + Qv + Zu + ε*, where *Y* is the observed vector for the phenotypic estimates of the traits; *β* is the fixed-effect vector (p × 1) other than the molecular marker effects and the population structure; *α* is the fixed-effect vector of the molecular markers; *v* is the fixed-effect vector from the population structure; *u* is the random-effect vector from the polygenic background effect; *X*, *W*, *Q*, and *Z* are the incidence matrixes from the associated *β*, *α*, *v*, and *u* parameters; and *ε* is the residual effect vector. A Manhattan plot was also generated to visualize the GWAS results over the entire genome, using the GWAS output from GAPIT [48]. The phenotypic variation explained by the model for a trait and a particular SNP was determined using stepwise regression implemented in the *lme4* R package. The SNP loci with significant association with the phenotypic traits were determined by the adjusted *p*-value using Bonferroni correction [49]. A quantile–quantile (Q–Q) plot was generated by plotting the negative logarithms (−log10) of the *p*-values of the SNPs against their expected *p*-values to fit the appropriateness of the GWAS model with the null hypothesis of no association, and to determine how well the models accounted for the population structure [50].

### 2.7. Identification of Putative Candidate Genes 

To annotate putative candidate genes for low-N tolerance, the physical positions of the significant SNPs were compared with the Maize B73 reference genome version 4 (RefGen_v4), available at the MaizeGDB database. The protein-coding genes in the vicinity of significant SNPs for the phenotypic traits were searched within the range of 1 Mb (500 kb upstream and downstream). Linkage disequilibrium (LD) was assessed among the significant SNPs, using the LDheatmap library [51]. The functions of the candidate genes were determined from the Universal Protein Resource (https://www.uniprot.org, accessed on 14 September 2021) and the European Molecular Biology Laboratory—European Bioinformatics Institute (https://www.ebi.ac.uk, accessed on 14 September 2021). 

## 3. Results

### 3.1. Evaluation of Phenotypic Traits

**Figure 1:**
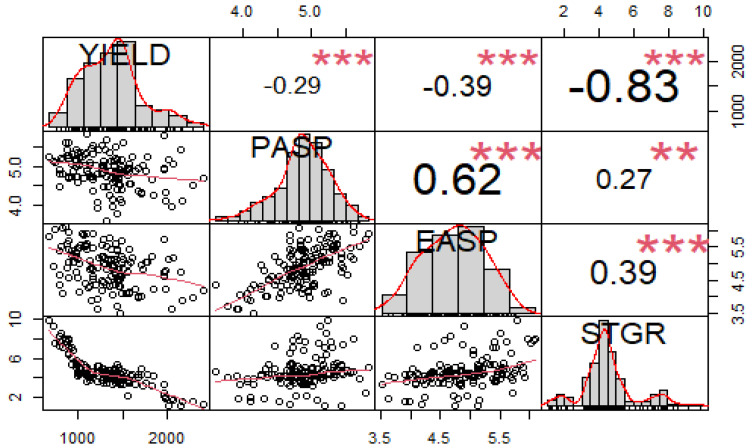
Correlation coefficients between low-N tolerance indicator traits and other agronomic traits of 169 early-maturing QPM inbred lines under low-N environments. YIELD = grain yield, PASP = plant aspect, EASP = ear aspect, EPP = ears per plant, STGR = stay-green characteristic. **, *** = Significant at 0.01 and 0.001 probability levels.

The analysis of variance (ANOVA) under the low-N environments revealed significant (*p* < 0.01) differences among the inbred lines (G) for all traits and environment (E) mean squares for all traits (Table 1). Significant variation (*p* < 0.01) was also observed for the Genotype × Environment interaction (GEI) mean squares for the measured traits, except for plant aspect, ear aspect, and stay-green characteristic. Broad sense heritability (H^2^) estimates ranged from 41% for plant aspect to 57% for grain yield. The phenotypic correlations among grain yield and other measured traits differed under the low-N environments (Figure 1). Grain yield had a significant and negative correlation with plant aspect (r = −0.29 **), ear aspect (r = −0.39 **), and stay-green characteristic (r = −0.80 **). A significant and positive correlation was recorded between the plant and ear aspects (r = 0.62 **). Similarly, the stay-green characteristic had a significant and positive correlation with both the plant and ear aspects (r = 0.25 **, r = 0.35 **). 

### 3.2. Population Structure and Genetic Diversity

**Figure 2:**
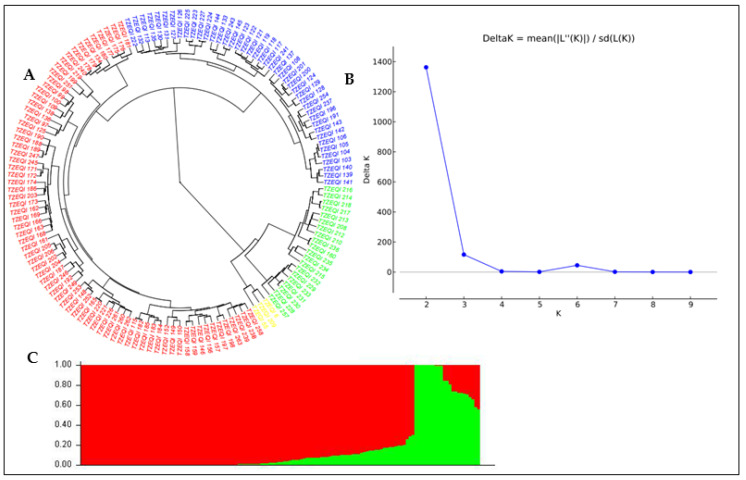
Population structure of the 140 QPM inbred lines. (**A**). Phylogeny tree showing the sub-populations based on the 7599 SNP markers. (**B**). Plot of the mean likelihood of delta K against the number of K groups. The highest peak, observed at K = 2, signifies the grouping of the inbred lines based on 7599 SNP markers into two groups. (**C**). Population structure originated from the STRUCTURE-based K = 2.

**Figure 3:**
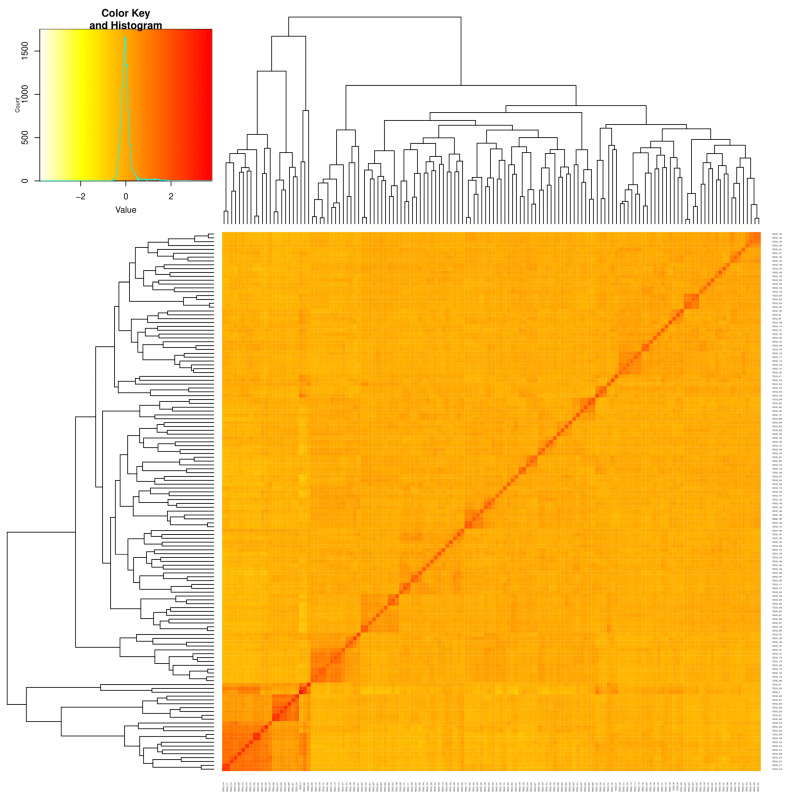
Heatmap showing pairwise Kinship matrix of the 140 QPM inbred lines.

Using the DArT sequencing technology, a total of 49,185 SNP markers were generated across the 140 QPM inbred lines. After quality filtering of the unmapped and duplicated markers, SNPs with missing values > 10% and MAF < 5% were removed, and 7599 SNPs were retained for the analysis. The MAF of the 7599 SNPs markers ranged from 0.01 to 0.5, with a mean of 0.21. The observed heterozygosity value ranged from 0.00 to 0.74, with a mean of 0.37. The expected heterozygosity value ranged from 0.1 to 0.5, with a mean of 0.30, while the polymorphic information content (PIC) ranged from 0.09 to 0.38, with a mean of 0.25 (Supplementary Figure S2). The population structure analysis of the QPM lines shows that the delta K values from the mean log-likelihood probabilities peaked at K = 2 (1362.62). At K = 2, 84% of the 140 QPM inbred lines were stratified into two sub-populations. The phylogenetic tree also revealed two major groups; a total of 117 inbred lines were grouped in Group 1, and 23 were placed in Group 2 (Figure 2 and Figure 3). The pairwise kinship matrix heatmap of the 140 inbred lines also revealed two major groups, with their familial relationships shown along the diagonal with a few large blocks of closely related individuals.

### 3.3. Linkage Disequilibrium (LD)

The linkage disequilibrium analysis revealed 17,982 loci pairs with approximately 35% (594) of the loci pairs in complete LD (R^2^ = 1). A pairwise linkage disequilibrium (LD) analysis between the 7599 SNPs across the 140 QPM inbred lines was performed to estimate the mapping resolution. The whole-genome LD decay peaked at an r^2^ of 0.47 and dropped at a distance of 250 kb, at an r^2^ of 0.32 (Supplementary Figure S3).

### 3.4. Genome-Wide Association and LD Analysis

Under a low-N environment, 40 SNPs were detected through the GWAS scan to be associated with four yield-related traits at a threshold of –log (p) = 3 (Table 2). The quantile–quantile (Q–Q) plots generated by plotting the negative logarithms (−log10) of the *p*-values against their expected *p*-values revealed associations between the phenotypes and the markers. The Q–Q plot revealed that more associations were found than was expected for grain yield and plant aspect than for ear aspect and stay-green characteristic.

#### 3.4.1. Grain Yield

**Figure 4:**
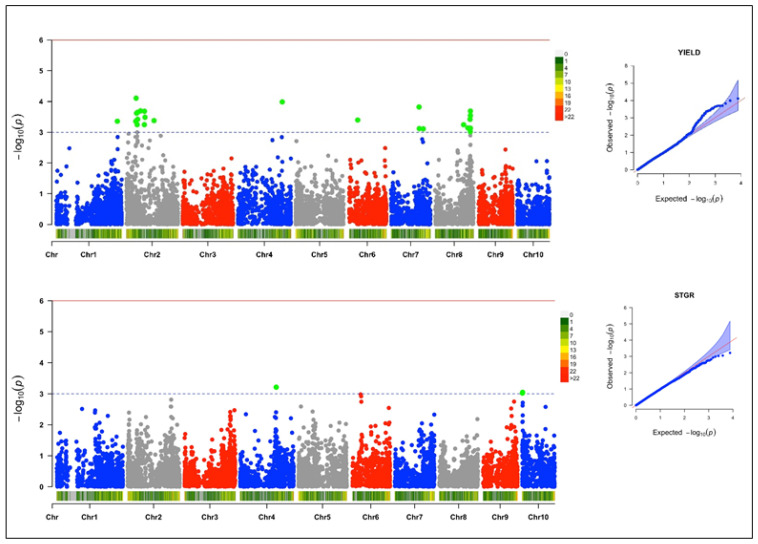
The Manhattan and Q–Q plots of the SNP-based association mappings for grain yield (YIELD) and stay-green characteristic (STGR) under low-N environments.

Twenty-four SNPs markers were found to be significantly associated with grain yield; these were scattered across six chromosomes (Figure 4). The MAF ranged from 0.02 to 0.37, and the trait variation explained by each SNP marker varied from 14% to 17%. The logarithm of the odd (LOD) values of the markers varied from 3.11 to 4.11. Of the 24 SNPs that were significantly associated with grain yield, 11 were mapped on chromosome 2, 7 on chromosome 8, 3 on chromosome 7 and 1 each on chromosomes 1, 4, and 6. The SNP markers S2_46273057 and S4_209096186 had the highest total explained phenotypic variance of 17%.

#### 3.4.2. Stay-Green Characteristic

Three SNP loci recorded a significant association with the stay-green characteristic under low-N environments (Figure 4). Of the three significant SNPs, one was found on chromosome 4, and the other two on chromosome 10. The LOD score of the markers ranged from 3.02 to 3.21, while the explained phenotypic variation ranged from 8% to 9%, with the MAF ranging from 0.24 to 0.50.

#### 3.4.3. Plant Aspect

**Figure 5:**
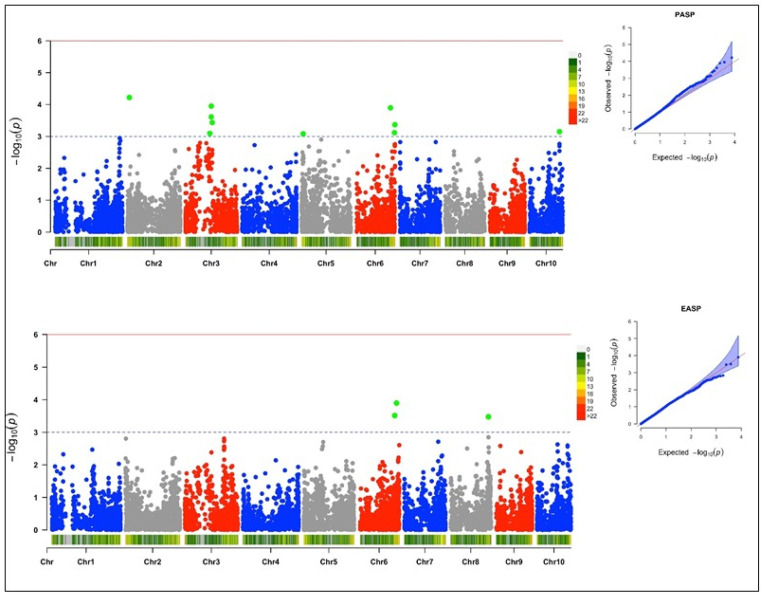
The Manhattan and Q–Q plots of the SNP-based association mappings for plant aspect (PASP) and ear aspect (EASP) under low-N environments.

Ten significant SNPs were found to be associated with plant aspect. Four of the significant SNPs were found on chromosome 3, three were found on chromosome 6, and one each was found on chromosomes 2, 5, and 10 (Figure 5). The LOD score and MAF of the SNPs ranged from 3.08 to 4.22, and from 0.09 to 0.34, respectively. The total explained phenotypic variation ranged from 12% to 16%.

#### 3.4.4. Ear Aspect

Three SNP loci were found to be significantly associated with ear aspect, with a LOD score ranging from 3.48 to 3.90 (Table 2) (Figure 5). Two SNPs were found on chromosome 6, while one significant SNP was found on chromosome 8. The MAF ranged from 0.06 to 0.19, with an explained phenotypic variation that ranged from 16% to 17%.

### 3.5. Candidate Gene Annotation

**Figure 6:**
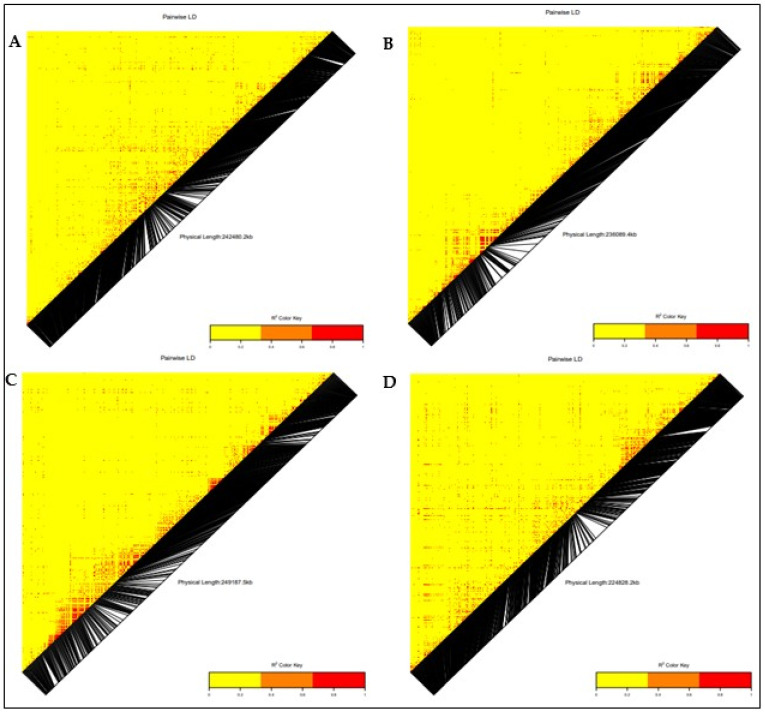
Heatmap LD haplotype blocks for different SNP markers located on different chromosomes. (**A**) Chromosome 2; (**B**) Chromosome 3; (**C**) Chromosome 4; and (**D**) Chromosome 5. The R^2^ colored key indicates the degree of significant association with the putative gene.

**Figure 7:**
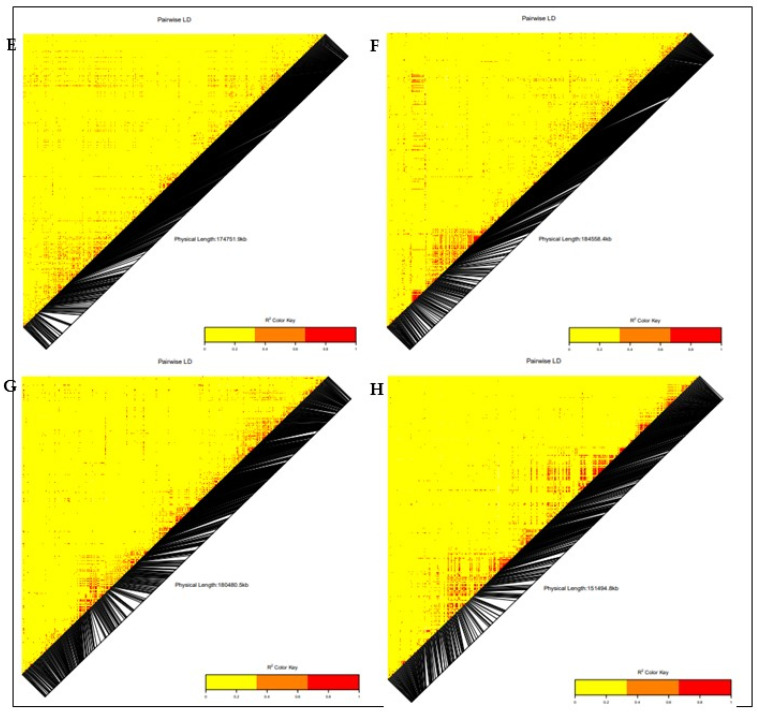
Heatmap LD haplotype blocks for different SNP markers located on different chromosomes. (**E**) Chromosome 6; (**F**) Chromosome 7; (**G**) Chromosome 8; and (**H**) Chromosome 10. The R^2^ colored key indicates the degree of significant associations with the putative gene.

The positions of significant SNPs in the maize genome were explored to identify potential protein-coding genes that were located in or close to the significant SNPs associated with the low-N adaptive traits from the maize genetic database (http://www.maizedb.org/, accessed on 14 September 2021). Putative genes within the significant SNP region were searched in a defined range of 1 MB at 500 kb (downstream and upstream). The functions of the genes associated with the identified SNPs were determined using the Universal Protein Resource (UniProt) and the European Molecular Biology Laboratory–European Bioinformatics Institute (EMBL–EBI) database (Table 3). The LD heatmap of the significant SNPs on chromosomes 2, 3, 4, 5, 6, 7, 8, and 10 revealed a high genetic correlation (0.2 to 1.0) between the SNPs in the vicinity of the peak adjacent to the putative gene (Figure 6 and Figure 7). On chromosome 2, the significant SNPs associated with grain yield were located close to 11 putative genes with known functions (Zeaxanthin epoxidase, Protein phosphatase, Protein auxin signaling F-box, high chlorophyll fluorescence 106, E3 ubiquitin-protein ligase UPL3, COP9 signalosome complex subunit 8, HVA22-like protein f, zinc finger protein CONSTANS-LIKE 1, putative RING zinc finger domain superfamily protein, MLO defense gene homolog 3, and SNARE-interacting protein keule). Two putative genes, namely dolichol-phosphate mannosyl transferase and E3 ubiquitin-protein ligase ATL31, were identified close to the significant SNP on chromosomes 1 and 7, respectively.

Five putative genes, namely SNF1-related protein kinase regulatory subunit γ-1, exocyst complex component EXO70B1, beclin-1-like protein, chitinase CLP, and photosystem II reaction center PSB28 protein, were identified close to significantly associated SNPs on chromosome 8, which displayed a moderately high correlation through the LD heatmap (Table 3). For the stay-green characteristic, two putative genes (Scarecrow-like protein 3 and disease resistance protein RGA5) were located in close proximity to the associated SNP on chromosome 10, with a moderately high correlation throughout the LD heatmap. Three putative genes (putative disease resistance protein RGA3, cystatin 3, and EID1-like F-box protein 3) were identified on chromosome 6, close to significant an SNP associated with plant aspect. Similarly, on chromosome 5, three putative genes (ATP synthase F1, delta subunit family protein, WD repeat-containing protein PCN, and GATA transcription factor 25) were each identified close to a significant SNP associated with plant aspect. On chromosome 10, a putative gene was identified in the vicinity of the significant SNP associated with plant aspect. Four putative genes (glutamate synthase 2 (NADH), chloroplastic, putative disease resistance protein RGA3, cystatin 3, EID1-like F-box protein 3, and protein auxin-regulated gene involved in organ size) were identified and were close to the significant SNPs associated with ear aspect on chromosomes 6 and 8.

## 4. Discussion

The significant genotypic variation that was observed among the 140 early-maturing white QPM inbred lines for grain yield and other studied traits suggested that considerable genetic variation existed among the inbred lines for low-N tolerance. The significant environment mean squares observed for the traits indicated the distinctness of the environments in discriminating among the inbred lines, under each research condition [16]. The relatively moderate heritability estimates recorded for grain yield and for the other traits implied that an appreciable degree of genetic variation existed for low-N tolerance in the QPM inbred lines. This implied that the detection of SNPs and of true association between a marker and a trait would be achievable for the genetic improvement of low-N tolerance through marker-assisted selection [21,52]. The significant and negative correlations observed between grain yield, stay-green characteristic, and plant and ear aspects implied that the simultaneous improvement of these traits would lead to an increase in grain yield. The correlation results also justified the inclusion of the measured traits in the selection index for grain yield improvement under low-N environments in SSA [16,32]. The average PIC value of 0.25 obtained in this study revealed the informativeness of the markers used in this study. Similar PIC values were reported by Eltaher et al. [53] and Adewale et al. [22]. The LOD score ranging from 3 to 4 suggests that the identified SNP markers could be considered as minor QTLs [50]. The high MAF (greater than 10%) recorded for the most significant SNPs indicated the positive detection power of the GWAS for eliminating spurious associations with rare alleles [50]. The results of the population structure analysis of the 140 inbred lines confirmed its importance in preventing false-positive associations [50].

The population structure of the inbred lines studied based on the delta K revealed two sub-populations, indicating moderate genetic variability within the population. The phylogeny analysis and the pairwise kinship matrix heatmap also revealed similar results, indicating their relevance in preventing spurious associations in the GWAS study [53,54]. The genome-wide LD decay at 250 kb and r^2^ = 0.32, as well as the existence of large marker pairs in LD, implied that significant marker–trait associations could be identified in the GWAS study [55,56]. The appropriateness of the model used for the GWAS study was confirmed by examining the quantile–quantile (Q–Q) plots to determine how well the models accounted for population structure. The Q–Q plots also revealed that the majority of points in the Q–Q plots aligned on the diagonal line for all of the traits, indicating that the population structure was well accounted for [50,52].

The genome-wide marker-trait association analyses for grain yield, stay-green characteristic, and plant and ear aspects identified 40 significant SNPs at the threshold of −log(p) = 3 on nine chromosomes (chromosomes 1, 2, 3, 4, 5, 6, 7, 8, and 10). Similar results were reported by Ertiro et al. [25] in a GWAS study on nitrogen use efficiency. The phenotypic variation of 8 to 17% in this study suggested that these SNPs would be useful for marker-assisted selection for low-N tolerance in SSA [22,50]. The identified SNP markers offer good targets for further validation analysis, due to their close proximity to 32 candidate genes regulating nitrogen biosynthesis and assimilation, plant defense mechanism, and growth and development. The significant SNPs (S2_46273057, S2_67297792, S2_85668156, S2_54250098, S2_48447873, and S2_88084334) associated with grain yield on chromosome 2 were close to 10 protein-coding putative genes (*GRMZM2G127139*, zeaxanthin epoxidase (*ZEP*); GRMZM2G015610, protein phosphatase; GRMZM5G848945, auxin signaling F-box (*AFB3*) protein; GRMZM2G049141, E3 ubiquitin-protein ligase (*UPL3*); GRMZM2G107588, *COP9* signalosome complex subunit 8 (*CSN8*); GRMZM2G157822, HVA22-like protein; GRMZM2G106108, zinc finger protein CONSTANS-like 1; GRMZM2G028543, putative RING zinc finger domain superfamily protein; GRMZM2G031331, *MLO* defense gene homolog 3; and GRMZM2G012942, *SNARE*-interacting protein keule). According to Schwarz et al. [55], zeaxanthin epoxidase catalyzes the conversion of zeaxanthin to violaxanthin, which is a key reaction for the biosynthesis of abscisic acid (ABA) and the xanthophyll cycle. ABA is an important plant hormone that regulates normal plant growth during vegetative development, stress responses, and other plant physiological processes, including leaf senescence, seed germination, and osmotic regulation [56,57]. Similarly, xanthophyll is important for photosynthetic energy conversion by maintaining the balance between light energy dissipation and the optimal utilization of photosynthesis [58,59]. Additionally, *AFB3* is a developmental protein in plants that regulates lateral and primary root development, and pollen development and maturation, as well as the cellular response to nitrate supply to plants [60,61]. *CSN8* is also a developmental protein and a subunit of the COP9 signalosome (CSN); it is an essential regulator of the ubiquitin conjugation pathway that plays an important role in plant growth and stress tolerance [62,63]. Chen et al. [64] reported HVA22 as an ABA-inducible gene that plays a role in stress tolerance and plant reproductive development. The *CONSTANS-like* gene family was reported by Tan et al. [65] and Wu et al. [66] as a transcription factor that is involved in the regulation of plant flowering time. Kim et al. [67] reported the *MLO* defense gene as being a negative modulator of plant disease resistance and leaf cell death, which is an indicator trait for low-N tolerance. On chromosome 8, significant SNPs (S8_166330750 and S8_164481914) associated with grain yield were identified close to protein-coding putative genes with identifiers such as GRMZM2G003493 (SNF1-related kinase regulatory subunit γ-1 protein), GRMZM2G003518 (exocyst complex component EXO70B1), GRMZM2G027857 (beclin-1-like protein), GRMZM2G005290 (chitinase CLP), and GRMZM2G005433 (photosystem II reaction center PSB28 protein). The SNF1-related protein kinase (SnRK) complex regulates the assimilation of nitrogen and leaf senescence in plants [68,69]. The exocyst complex component EXO70B1 plays a role in plant defense responses to biotic and abiotic stresses [70,71]. Beclin-1-like protein is an autophagy-related protein that is required for plant growth and development and pollen development; it is also involved in the cellular response to nitrogen stress [72]. According to Coe et al. [73], photosystem II PSB28 is a membrane protein that executes the initial reaction of photosynthesis in higher plants, which is a requirement for normal plant development. The E3 ubiquitin-protein ligase *ATL31*, found near a significant SNP (S7_132400339) associated with grain yield, was reported by Sato et al. [74] to be involved in the cellular response to nitrogen levels during plant growth. Similarly, on chromosome 1, a putative protein gene encoding dolichol-phosphate mannosyltransferase was found near a grain yield-associated SNP. Jadid et al. [75] reported dolichol-phosphate mannosyltransferase to play a role in plant development and sensitivity to ammonium stress, which is a preferred nitrogen-containing nutrient for plant growth that is converted to nitrite (NO_2_) and nitrate (NO_3_) before it is used by plants.

A putative gene (Scarecrow-like protein and disease resistance protein RGA5) found near an associated SNP (S10_1417870) with a stay-green characteristic on chromosome 10, is a transcription factor involved in plant leaf development, and also promotes the biosynthesis of ABA [76]. The RGA5 resistance protein have been reported by Césari et al. [77] to be involved in the negative regulation of cell death, which implies that it could lead to delayed senescence in plants. On chromosome 6, a significant SNP (S6_159734917) associated with plant and ear aspects was found in close proximity to three protein-coding putative genes with the identifiers GRMZM2G440849 (putative disease resistance protein RGA3), GRMZM2G440968 (cystatin 3), and GRMZM2G389301 (EID1-like F-box protein 3). The significant SNP S6_159734917, identified for both plant and ear aspects, implied a possible simultaneous improvement in the two traits when using the same set of genomic markers. The RGA proteins, as reported by Sekhwal et al. [78], are resistance proteins that trigger the plant’s defense system against diseases such as leaf rust, Fusarium wilt, and corn leaf blight. Koops et al. [79] reported the EDL3 F-box protein to be involved in the regulation of abscisic acid signaling, which is a plant hormone that regulates plant growth and development.

The SNP (S5_8518748) on chromosome 5 associated with plant aspect was found to be close to the putative genes GRMZM2G401040 (ATP synthase F1, delta subunit family protein), GRMZM2G065822 (WD repeat-containing protein PCN), and GRMZM2G065896 (GATA transcription factor 25). Xiang et al. [80] reported that the WD repeat gene is required for leaf development, root and shoot meristem growth, leaf formation, and plant organ development in Arabidopsis thaliana. The GATA transcription factor is an evolutionary transcription regulator, with the G-A-T-A core sequence being involved in the regulation of chlorophyll biosynthesis, nitrogen compound metabolism, and plant organ senescence [81,82]. On chromosome 6, a putative gene, GRMZM2G375064 (glutamate synthase) was found near an SNP (S6_167701917) that is significantly associated with ear aspect. Lancien et al. [83] and Tamura et al. [84] reported glutamate synthase to be involved in the nitrogen metabolism pathway, as well as its assimilation in seedling roots. However, a similar gene was reported by Morosini et al. [85], on chromosome 10. On chromosome 8, a SNP (S8_169668528) was found to be close to putative genes (GRMZM2G171996) encoding auxin-regulated protein; a developmental protein that was reported by Hu et al. [86] and Wang et al. [87] to be involved in the regulation of plant organ development. The putative genes identified in the present study possess reliable information for functional gene studies, and there is a need for their further validation for their use in the improvement of low-N tolerance in maize.

## 5. Conclusions

Forty (40) significant SNPs were identified to be associated with the four measured traits under low-N conditions. SNP S6_159734917 was identified for both plant and ear aspects, confirming the positive correlation between the traits, and the possibility of simultaneous improvement of the two traits using the same set of genomic markers. Significant SNPs were found in the vicinity of 32 protein-coding putative genes. Eighteen of the genes were associated with grain yield, while seven and five were found to be close to the SNPs associated with plant and ear aspects, respectively. The genes *GRMZM2G127139, GRMZM5G848945, GRMZM2G031331, GRMZM2G003493, GRMZM2G067964, and GRMZM2G180254*, found on chromosomes 1, 2, 8 and 10, were involved in cellular nitrogen assimilation and biosynthesis, and they may be invaluable when breeding for low-N tolerant maize. The genes identified with different plant biosynthetic mechanisms applicable to maize under low-N could be useful for functional gene studies to clarify the genetic mechanisms underlying low-N tolerance. Additionally, the identified significant loci require further validation. The identified genes and the associated SNPs after validation could be used for marker-assisted selection, to accelerate genetic gains in the improvement of maize for low-N tolerance in SSA.

## Figures and Tables

**Table 1:** Mean squares derived from the analysis of variance table for grain yield and other agronomic traits of 169 early-maturing QPM inbred lines under low-N conditions at Mokwa 2019 and 2020 growing seasons.

SV	DF	YIELD	PASP	EASP	STGR
ENV	1	28,069,163.12 **	116.81 **	130.65 **	276.07 **
Block (ENV*Rep)	48	519,164.92 **	1.69 **	2.66 **	2.27 **
Rep (ENV)	2	314,250.52	8.35 **	11.94 **	16.55 **
Genotypes	168	438,971.11 **	0.70 **	1.28 **	2.23 **
ENV*Genotypes	168	456,508.9 **	0.47	0.62	1.31
Error	288	241,768.5	0.41	0.60	1.07
H^2^		0.57	0.41	0.45	0.43

*, ** = Significant at 0.05 and 0.01 probability levels, respectively; ENV = environment; Rep = replication; YIELD = Grain yield; PASP = plant aspect; EASP = ear aspect; EPP = ears per plant; STGR = stay-green characteristic.

**Table 2:** Significant SNP associations with low-N adaptive traits of 140 QPM inbred lines evaluated under low-N conditions at Mokwa during 2019 and 2020 growing seasons.

Traits	SNP	Chr	Position	*p*-Value	MAF	Marker R^2^	LOD
Grain yield	S2_46273057	2	46273057	7.8 × 10^−5^	0.05	0.17	4.11
	S4_209096186	4	209096186	1.0 × 10^−4^	0.20	0.17	3.99
	S7_131622616	7	131622616	1.5 × 10^−4^	0.08	0.16	3.82
	S2_67297792	2	67297792	2.0 × 10^−4^	0.31	0.16	3.69
	S8_166330750	8	166330750	2.1 × 10^−4^	0.29	0.16	3.69
	S2_85668156	2	85668156	2.1 × 10^−4^	0.04	0.16	3.68
	S2_54250098	2	54250098	2.2 × 10^−4^	0.12	0.16	3.65
	S2_48447873	2	48447873	2.4 × 10^−4^	0.05	0.16	3.62
	S8_166202087	8	166202087	2.9 × 10^−4^	0.29	0.16	3.54
	S8_166330684	8	166330684	2.9 × 10^−4^	0.28	0.16	3.54
	S2_88084334	2	88084334	3.3 × 10^−4^	0.06	0.15	3.49
	S2_53491979	2	53491979	3.8 × 10^−4^	0.06	0.15	3.42
	S8_164481914	8	164481914	3.8 × 10^−4^	0.21	0.15	3.42
	S6_45087496	6	45087496	4.0 × 10^−4^	0.02	0.15	3.40
	S2_130453666	2	130453666	4.2 × 10^−4^	0.04	0.15	3.38
	S1_287891383	1	287891383	4.4 × 10^−4^	0.37	0.15	3.36
	S2_44598915	2	44598915	4.4 × 10^−4^	0.07	0.15	3.36
	S2_51434022	2	51434022	5.7 × 10^−4^	0.04	0.15	3.24
	S2_85053187	2	85053187	5.7 × 10^−4^	0.04	0.15	3.24
	S8_134334368	8	134334368	5.7 × 10^−4^	0.05	0.15	3.24
	S8_154518069	8	154518069	7.3 × 10^−4^	0.14	0.14	3.13
	S8_166462404	8	166462404	7.4 × 10^−4^	0.30	0.14	3.13
	S7_132400339	7	132400339	7.6 × 10^−4^	0.08	0.14	3.12
	S7_151236665	7	151236665	7.8 × 10^−4^	0.19	0.14	3.11
Stay green characteristic	S4_168465704	4	168465704	6.1 × 10^−4^	0.24	0.09	3.21
	S10_2268677	10	2268677	8.9 × 10^−4^	0.24	0.08	3.05
	S10_1417870	10	1417870	9.4 × 10^−4^	0.50	0.08	3.02
Plant aspect	S2_10153860	2	10153860	6.0 × 10^−5^	0.16	0.16	4.22
	S3_119115762	3	119115762	1.1 × 10^−4^	0.10	0.15	3.95
	S6_159734917	6	159734917	1.2 × 10^−4^	0.19	0.15	3.90
	S3_119024277	3	119024277	2.4 × 10^−4^	0.12	0.14	3.61
	S3_123440369	3	123440369	3.7 × 10^−4^	0.10	0.13	3.44
	S6_179917351	6	179917351	4.3 × 10^−4^	0.09	0.13	3.37
	S10_136641842	10	136641842	7.1 × 10^−4^	0.34	0.12	3.15
	S6_177721271	6	177721271	7.6 × 10^−4^	0.15	0.12	3.12
	S3_112551813	3	112551813	8.0 × 10^−4^	0.09	0.12	3.10
	S5_8518748	5	8518748	8.3 × 10^−4^	0.10	0.12	3.08
Ear aspect	S6_167701917	6	167701917	1.3 × 10^−4^	0.06	0.17	3.90
	S6_159734917	6	159734917	3.1 × 10^−4^	0.19	0.16	3.51
	S8_169668528	8	169668528	3.3 × 10^−4^	0.11	0.16	3.48

**Table 3:** Candidate genes and their functions for each significant SNP associated with low-N adaptive traits.

Traits	Chr	Position	Gene ID	Encoding Products	Functions
Grain yield	2	46273057	*GRMZM2G127139*	Zeaxanthin epoxidase	Biosynthesis of abscisic acid
			*GRMZM2G015610*	Protein phosphatase	Unknown function
	1	287891383	*GRMZM2G067964*	Dolichol-phosphate mannosyltransferase	Plant growth and development
	2	67297792	*GRMZM5G848945*	Protein AUXIN SIGNALING F-BOX	Primary and lateral root development
			*GRMZM5G898735*	high chlorophyll fluorescence 106	Photosynthesis
	2	85668156	*GRMZM2G049141*	E3 ubiquitin-protein ligase UPL3	Unknown function
	2	54250098	*GRMZM2G107588*	COP9 signalosome complex subunit 8	Plant growth and stress tolerance
			*GRMZM2G157822*	HVA22-like protein f	Plant reproductive development
	2	48447873	*GRMZM2G106108*	zinc finger protein CONSTANS-LIKE 1	Plant flowering time
			*GRMZM2G028543*	putative RING zinc finger domain superfamily protein	Plant growth and development
			*GRMZM2G031331*	mlo defense gene homolog 3	Plant disease resistance and leaf cell death
	2	88084334	*GRMZM2G012942*	SNARE-interacting protein KEULE	
	7	132400339	*GRMZM2G082653*	E3 ubiquitin-protein ligase ATL31	Cellular response to nitrogen levels
	8	166330750	*GRMZM2G003493*	SNF1-related protein kinase regulatory subunit γ-1	Assimilation of nitrogen in plants
			*GRMZM2G003518*	exocyst complex component EXO70B1	Plant defense response to stress
			*GRMZM2G027857*	beclin-1-like protein	Cellular response to nitrogen
	8	164481914	*GRMZM2G005290*	chitinase CLP	Root and shoot development
			*GRMZM2G005433*	photosystem II reaction center PSB28 protein	Photosynthesis in plants
Stay Green Characteristic	10	1417870	*AC198366.3_FG004*	Scarecrow-like protein 3	Plant leaf development
			*GRMZM2G180254*	disease resistance protein RGA5	Plant cell death
Plant Aspect	6	159734917	*GRMZM2G440849*	putative disease resistance protein RGA3	Disease resistance in plant
			*GRMZM2G440968*	cystatin 3	Unknown function
			*GRMZM2G389301*	EID1-like F-box protein 3	Plant growth and development
	10	136641842	*GRMZM2G169645*	putative RING zinc finger domain superfamily protein	Unknown function
	5	8518748	*GRMZM2G401040*	ATP synthase F1, delta subunit family protein	Energy conversion in photosynthesis
			*GRMZM2G065822*	WD repeat-containing protein PCN	Leaf formation and development
			*GRMZM2G065896*	GATA transcription factor 25	Chlorophyll biosynthesis
Ear Aspect	6	167701917	*GRMZM2G375064*	glutamate synthase 2 (NADH), chloroplastic	Nitrogen metabolism
	6	159734917	*GRMZM2G440849*	putative disease resistance protein RGA3	Plant resistance to diseases
			*GRMZM2G440968*	cystatin 3	Unknown function
			*GRMZM2G389301*	EID1-like F-box protein 3	Regulates plant growth and development
	8	169668528	*GRMZM2G171996*	protein auxin-regulated gene involved in organ size	Plant organ development

## Data Availability

The DArTseq datasets used in the present study have been deposited at the IITA CKAN repository http://data.iita.org/, accessed on 27 March 2022.

## Supplementary Materials

The following supporting information can be downloaded at: https://www.mdpi.com/article/10.3390/genes13050826/s1, Figure S1: Extraction of the early-maturing QPM inbred lines using the pedigree selection method; Figure S2: Summary statistics of the SNP 7599 markers; Figure S3: Linkage disequilibrium decay; Table S1: List of 140 early-maturing QPM inbred lines.
