# Supplementary material for: Genome-Wide Association Analysis Reveals Genetic Architecture and Candidate Genes Associated with Grain Yield and Other Traits under Low Soil Nitrogen in Early-Maturing White Quality Protein Maize Inbred Lines
Source: Genes (Basel). 2022 May 5;13(5):826. doi: 10.3390/genes13050826 (PMC9141126; doi:10.3390/genes13050826)
Supplement: Supplementary file 1 [file genes-13-00826-s001.zip › Figure S3- Development of Mapping Population.pdf]

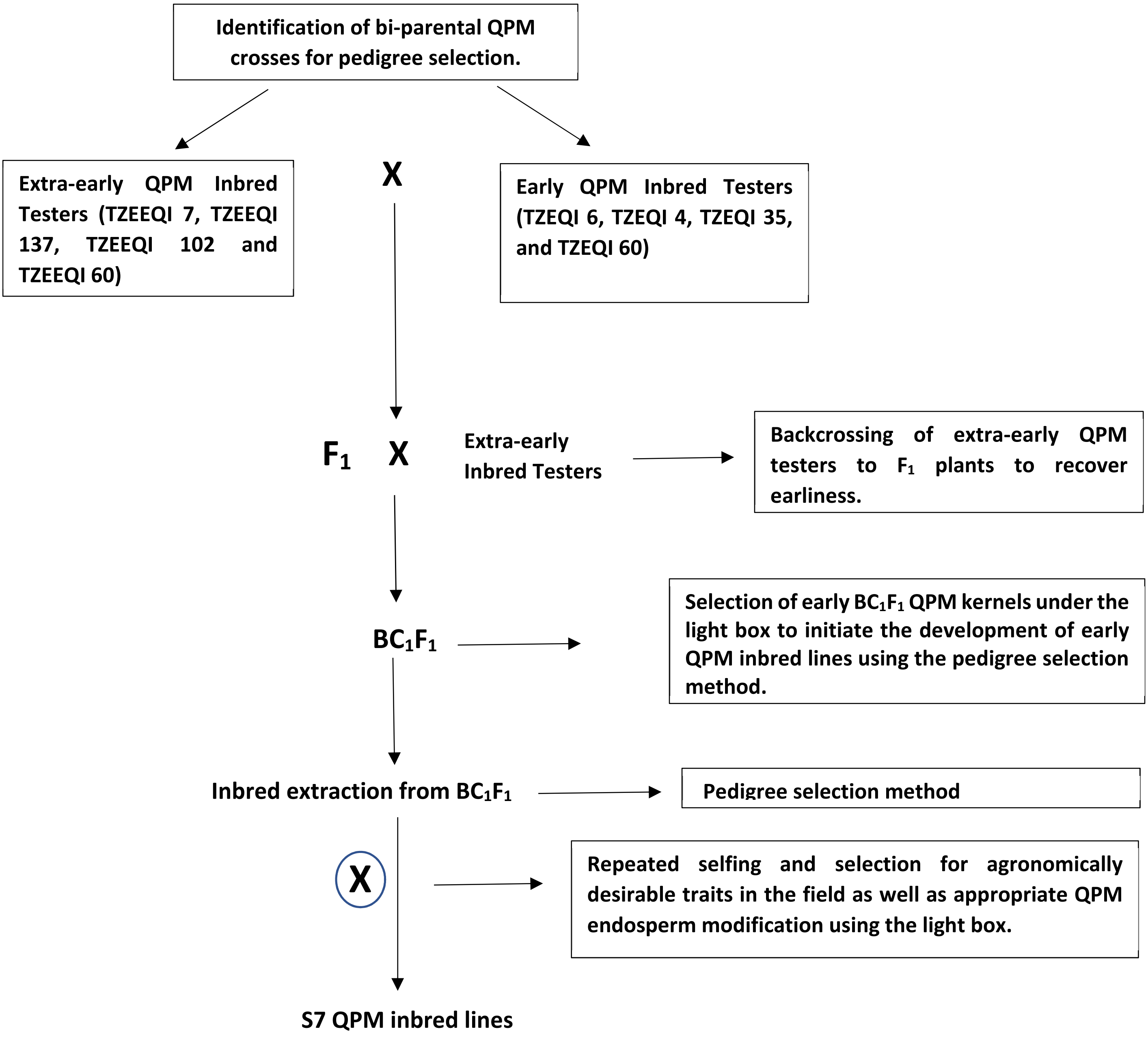

Figure S3. Extraction of the early maturing QPM inbred lines using the pedigree selection method.
