# Supplementary figures and images for: Genome-Wide Association Analysis Reveals Genetic Architecture and Candidate Genes Associated with Grain Yield and Other Traits under Low Soil Nitrogen in Early-Maturing White Quality Protein Maize Inbred Lines
Source: Genes (Basel). 2022 May 5;13(5):826. doi: 10.3390/genes13050826 (PMC9141126; doi:10.3390/genes13050826)

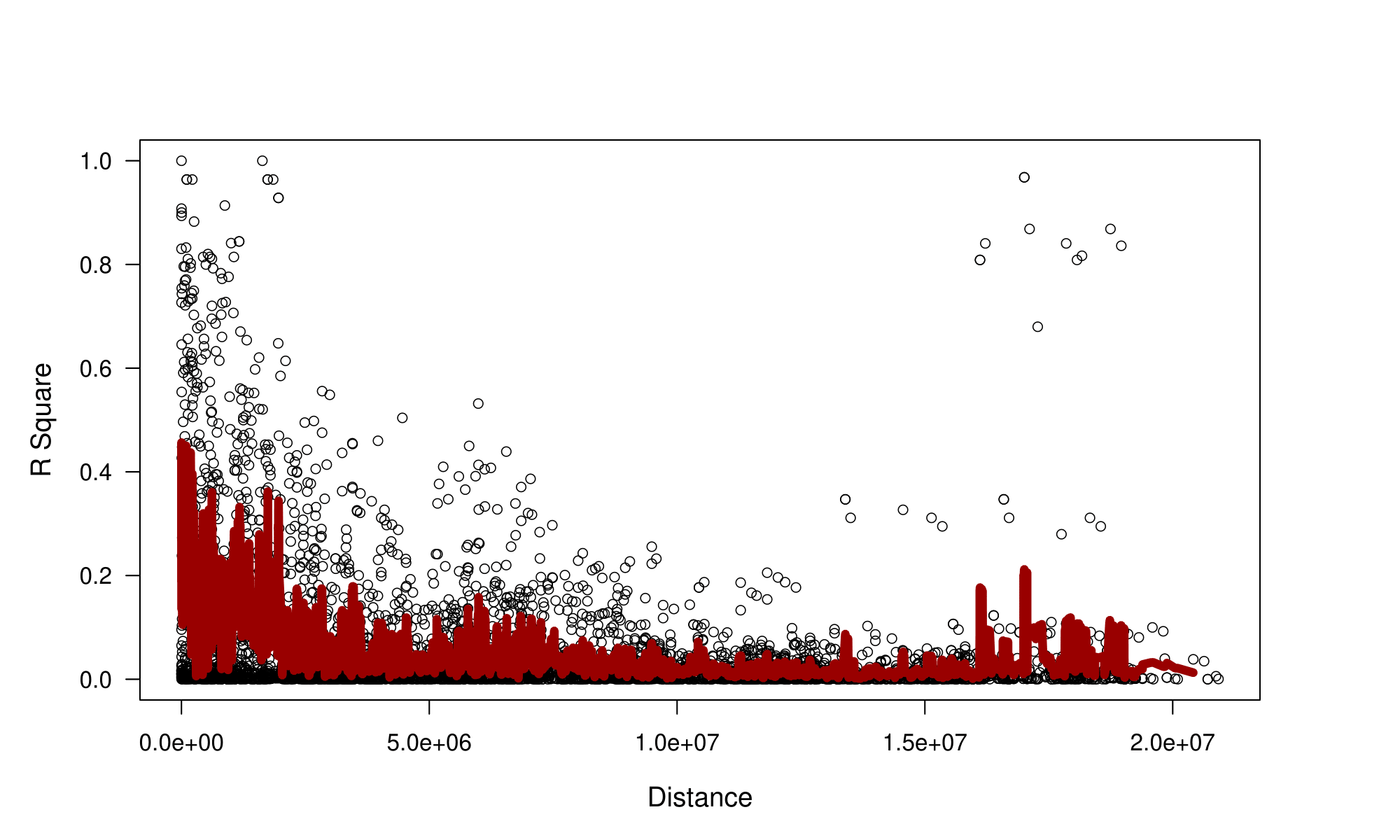

Supplement: Supplementary file 1 [file genes-13-00826-s001.zip › Figure S1- LD DECAY.png]

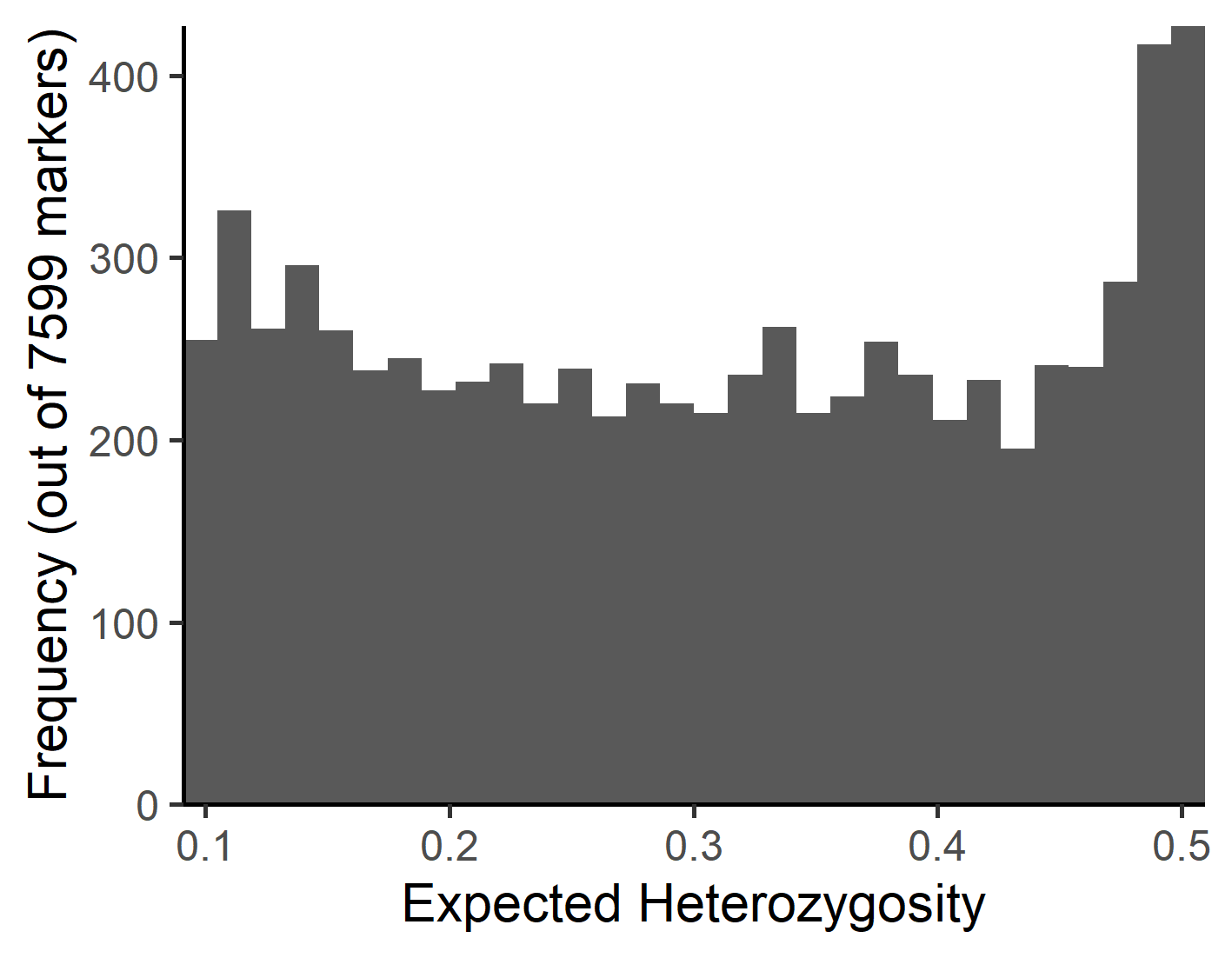

Supplement: Supplementary file 1 [file genes-13-00826-s001.zip › Figure S2- Summary Statistics/Expected Heterozygosity.tiff]

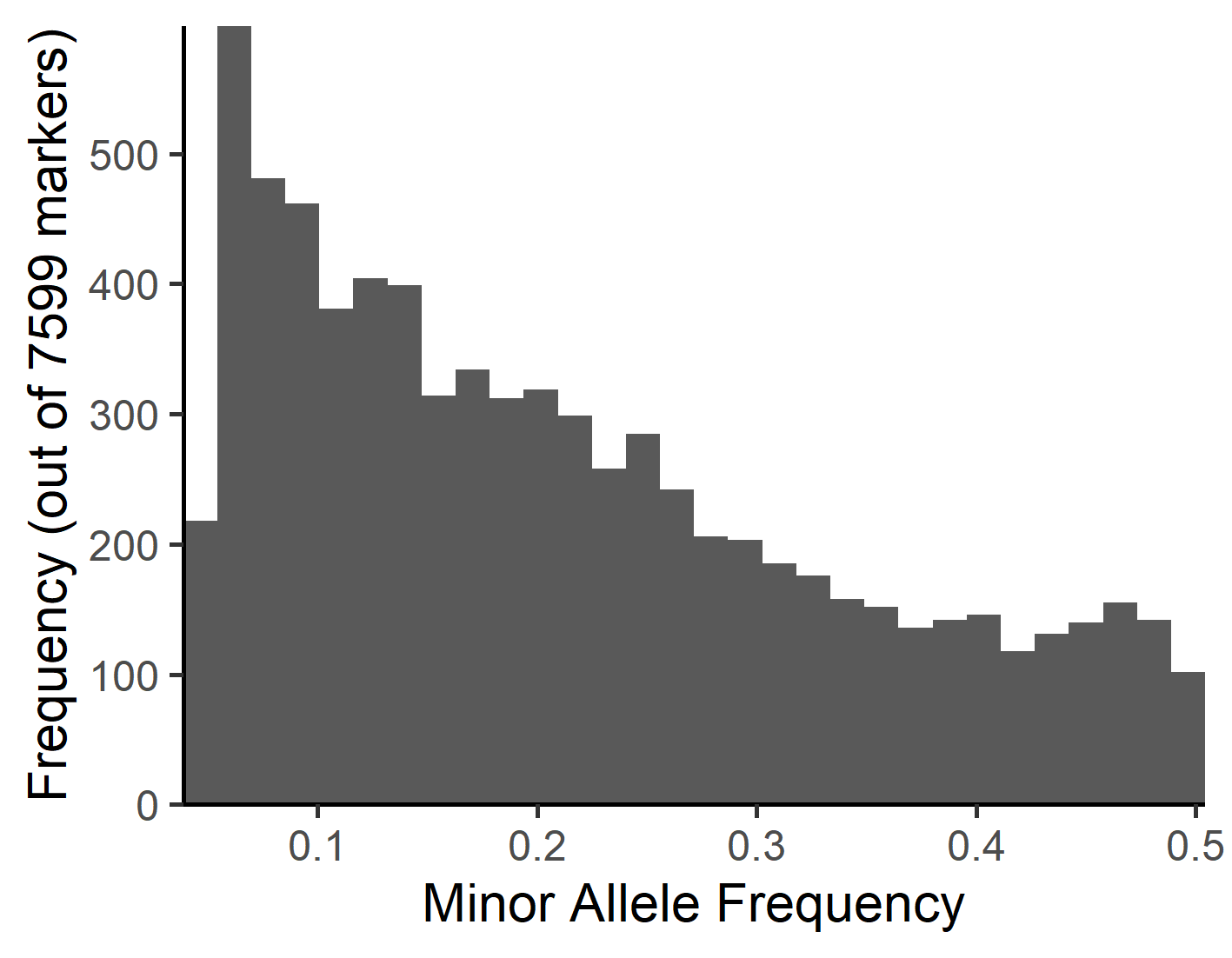

Supplement: Supplementary file 1 [file genes-13-00826-s001.zip › Figure S2- Summary Statistics/MAF.tiff]

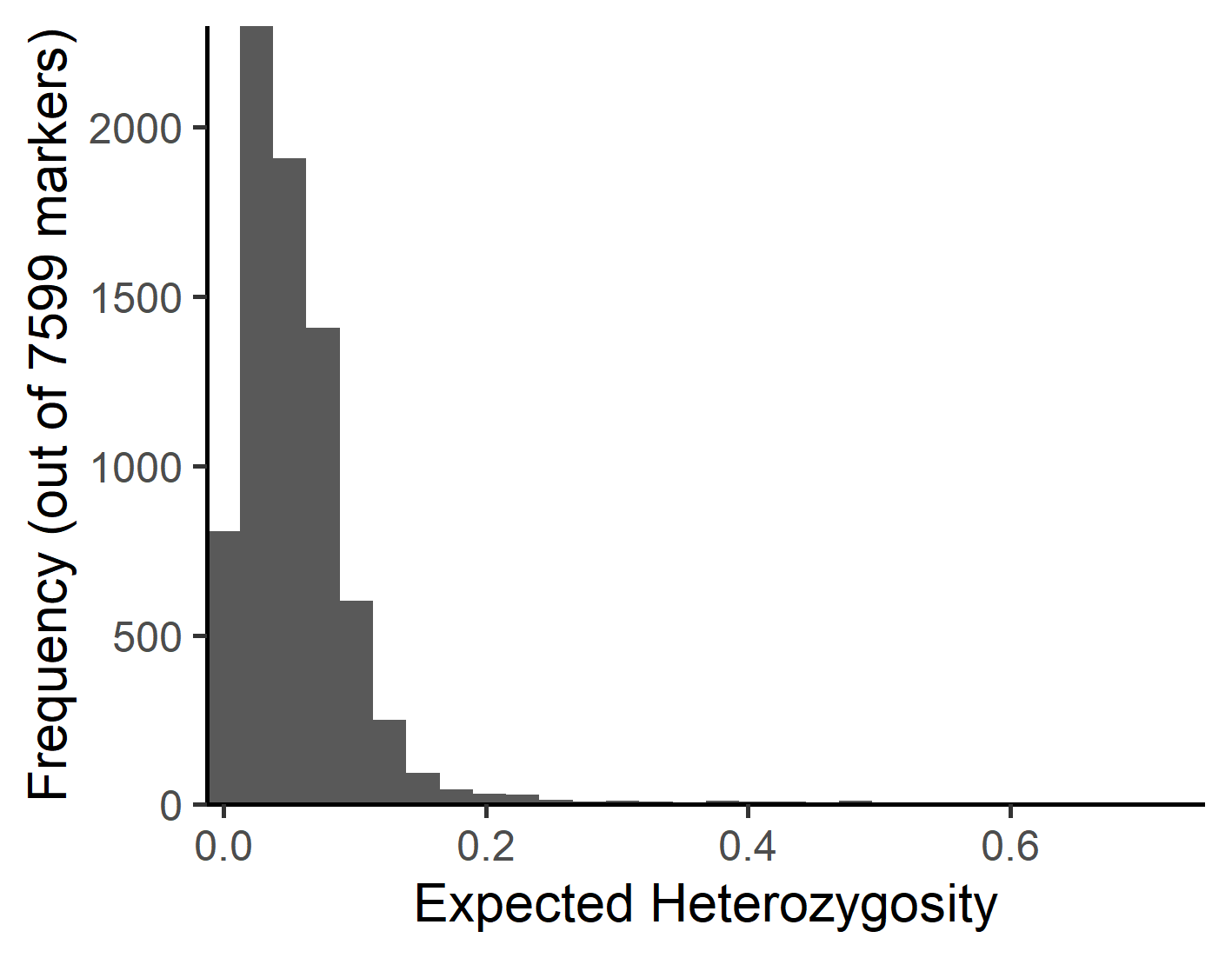

Supplement: Supplementary file 1 [file genes-13-00826-s001.zip › Figure S2- Summary Statistics/Observed Heterozygosity.tiff]

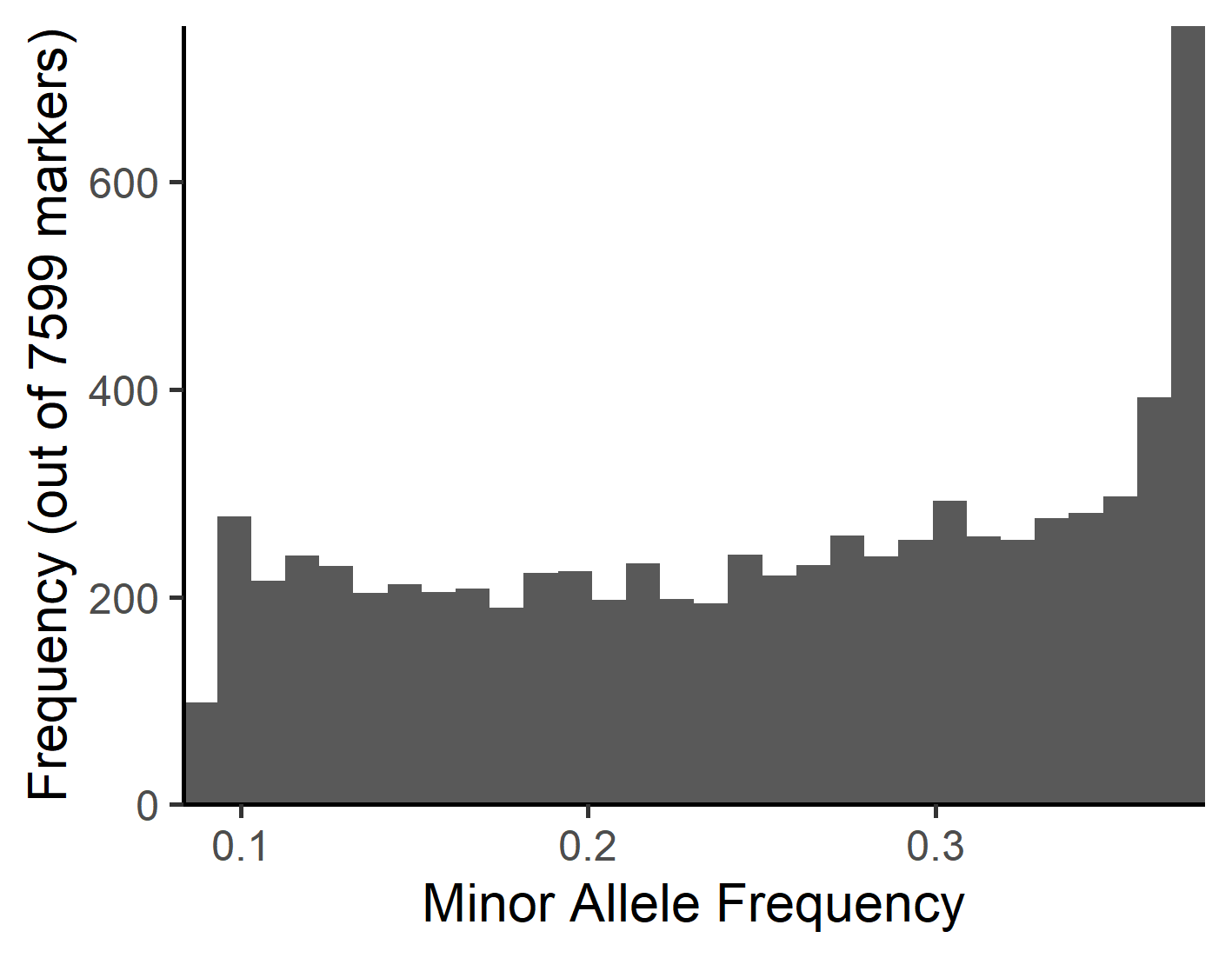

Supplement: Supplementary file 1 [file genes-13-00826-s001.zip › Figure S2- Summary Statistics/Polymorphic Information Content.tiff]
